# Development and Validation of a Predictive Scoring System for In-hospital Death in Patients With Intra-Abdominal Infection: A Single-Center 10-Year Retrospective Study

**DOI:** 10.3389/fmed.2021.741914

**Published:** 2021-11-12

**Authors:** Gaici Xue, Hongyi Liang, Jiasheng Ye, Jingjing Ji, Jianyu Chen, Bo Ji, Zhifeng Liu

**Affiliations:** ^1^Department of Neurosurgery, General Hospital of Southern Theatre Command of People's Liberation Army of China (PLA), Guangzhou, China; ^2^Department of Clinical Pharmacy, General Hospital of Southern Theatre Command of People's Liberation Army of China (PLA), Guangzhou, China; ^3^Department of Critical Care Medicine, General Hospital of Southern Theatre Command of People's Liberation Army of China (PLA), Guangzhou, China; ^4^Department of Pediatric Internal Medicine, Guangzhou Women and Children's Medical Center, Guangzhou, China

**Keywords:** intra-abdominal infection, in-hospital death, risk factors, predictive scoring system, bootstrapping sample

## Abstract

**Objective:** To develop and validate a scoring system to predict the risk of in-hospital death in patients with intra-abdominal infection (IAI).

**Materials and Methods:** Patients with IAI (*n* = 417) treated at our hospital between June 2010 and May 2020 were retrospectively reviewed. Risk factors for in-hospital death were identified by logistic regression analysis. The regression coefficients of each risk factor were re-assigned using the mathematical transformation principle to establish a convenient predictive scoring system. The scoring system was internally validated by bootstrapping sample method.

**Results:** Fifty-three (53/417, 12.7%) patients died during hospitalization. On logistic regression analysis, high APACHE II score (*P* = 0.012), pneumonia (*P* = 0.002), abdominal surgery (*P* = 0.001), hypoproteinemia (*P* = 0.025), and chronic renal insufficiency (*P* = 0.001) were independent risk factors for in-hospital death. On receiver operating characteristic curve analysis, the composite index combining these five risk factors showed a 62.3% sensitivity and 80.2% specificity for predicting in-hospital death (area under the curve: 0.778; 95% confidence interval: 0.711–0.845, *P* < 0.001). The predictive ability of the composite index was better than that of each independent risk factor. A scoring system (0–14 points) was established by re-assigning each risk factor based on the logistic regression coefficient: APACHE II score (10–15 score, 1 point; >15 score, 4 points); pneumonia (2 points), abdominal surgery (2 points), hypoproteinemia (2 points), and chronic renal insufficiency (4 points). Internal validation by 1,000 bootstrapping sample showed relatively high discriminative ability of the scoring system (C-index = 0.756, 95% confidence interval: 0.753–0.758).

**Conclusions:** The predictive scoring system based on APACHE II score, pneumonia, abdominal surgery, hypoproteinemia, and chronic renal insufficiency can help predict the risk of in-hospital death in patients with IAI.

## Introduction

Intra-abdominal infection (IAI) is a common cause of admission to the intensive care unit, and is the second most common cause of septic shock after respiratory tract infection (1, 2). Treatment of IAI is inherently challenging because of its widespread origin, variety of pathogens, and the tendency to lead to sepsis and multiorgan failure. IAI is an important cause of non-traumatic death of hospitalized patients, with mortality rates as high as 20–29.1% (3–5).

The poor prognosis of patients with IAI, especially death, is the core concern of family members and clinicians. However, the personal experience of clinicians alone is not sufficient to provide an accurate clinical prognosis for patients and their families. Excessively pessimistic expectation of the patients' prognosis often leads to premature withdrawal of treatment by the family members. Development of objective measures for prognostic assessment of patients with IAI in clinical practice may help inform better treatment decision-making that is most conducive to the situation of individual patients and their care givers. This study aimed to provide a theoretical basis for accurate prognostic assessment of IAI patients by retrospectively analyzing the IAI cases admitted to our center over the past 10 years. Independent risk factors were identified and a scoring system to predict the risk of in-hospital death was developed. A simple scoring system for predicting the risk of in-hospital death of IAI patients was also established by converting the risk factors through use of mathematical principles.

## Materials and Methods

### Case Selection

The baseline data and clinical outcomes of IAI patients treated between June 2010 and May 2020 at our institution were retrospectively collected.

**Figure 1:**
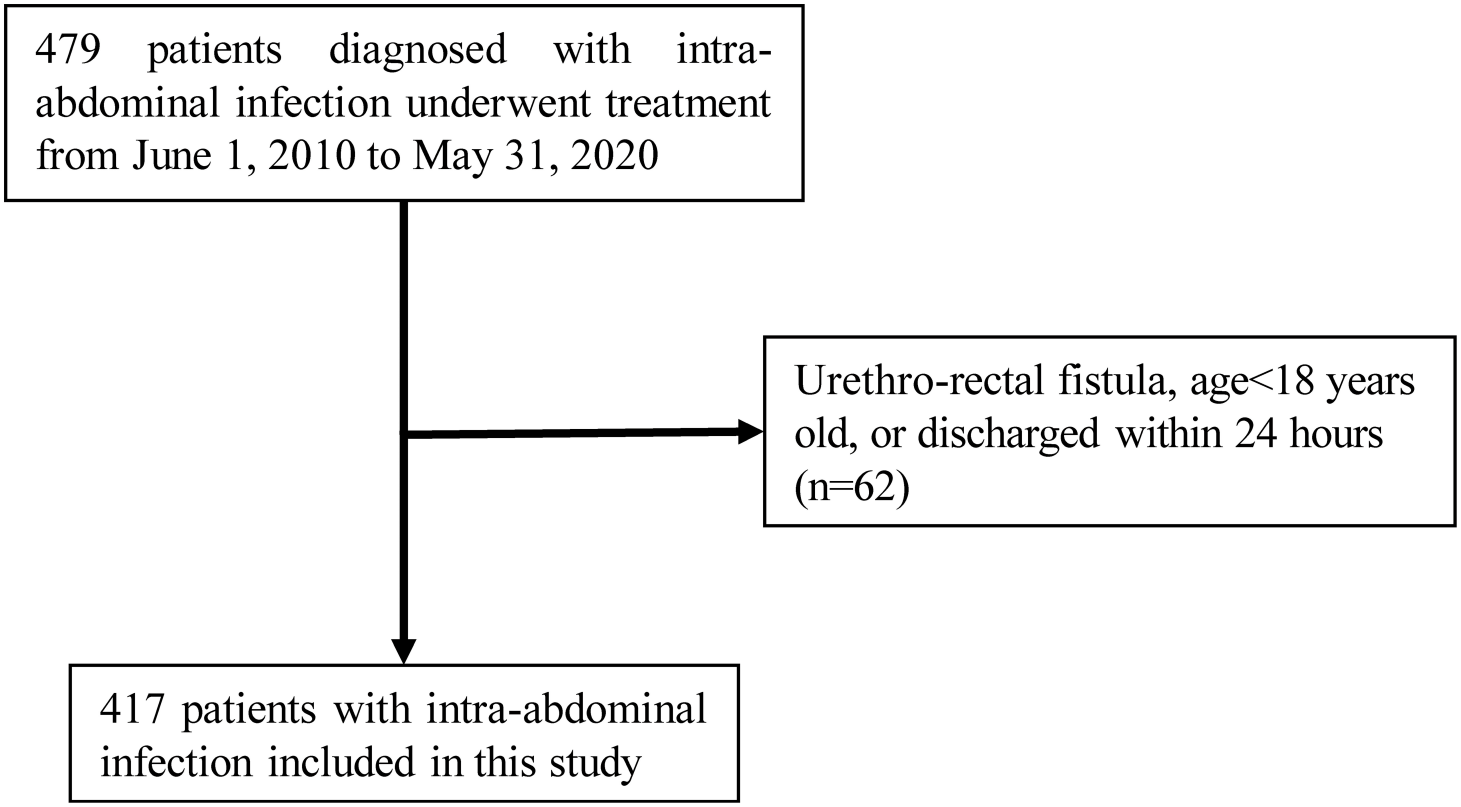
Flow diagram of patient selection according to the inclusion and exclusion criteria.

Inclusion criteria: adult patients aged over 18 years with uncomplicated or complicated abdominal infection, i.e., clinical manifestations of abdominal pain or systemic inflammatory response, with foci of infection confined within the wall of the gastrointestinal tract or extending to the peritoneal cavity from the cavernous organs of the abdominal cavity, accompanied by peritonitis or intra-abdominal abscess formation (6). Exclusion Criteria: (1) patient discharged in <24 h; (2) incomplete medical records. A total of 417 IAI patients qualified the selection criteria (Figure 1). This study was approved by the Ethics Committee of the General Hospital of the Southern Theater Command [Number: Hospital Ethics (2020)-8]. The requirement for informed consent of patients was waived off for this study in accordance with the national legislation and the institutional requirements.

### Definitions of Risk Factors for Univariate Analysis and Logistic Regression Analysis

The followed potential risk factors for in-hospital death were included in the analysis: patient's age, sex, body mass index (BMI); Acute Physiology and Chronic Health Evaluation II (APACHE II) score (evaluated at the time of diagnosis of IAI); Sequential Organ Failure Assessment (SOFA) score (evaluated at the time of diagnosis of IAI); history of underlying diseases (hypertension, diabetes, coronary heart disease); concomitant diseases [pneumonia (imaging signs of pulmonary inflammation affecting at least one lobe), cirrhosis (defined as Child-Pugh B or C), chronic renal insufficiency (defined as previous diagnosis of chronic kidney disease stage 2–5 prior to admission), cancer (defined as malignant tumor with definite pathological or imaging diagnosis before admission)]; hypoproteinemia (defined as serum total protein <60 g/L or albumin <35 g/L); organ transplantation (within 30 days); abdominal surgery (defined as abdominal surgery within the preceding 30 days due to trauma, tumor, inflammation, etc.); type of antibiotics used (broad-spectrum antibiotics alone, nitroimidazole drugs alone, or combination of nitroimidazole drugs with broad-spectrum antibiotics).

### Statistical Analysis

#### Determination of Independent Risk Factors and Development of the Predictive Scoring System

Statistical analyses were performed using SPSS 25.0 software and R version 3.6.3. Categorical variables were presented as frequency (percentage) while continuous variables were presented as mean ± standard deviation (SD) or median (the first quartile, the third quartile). Univariate analysis was performed using two independent samples *t-*test, Pearson Chi-squared test, Fisher exact test, or non-parametric test to initially screen the risk factors for in-hospital death. Factors associated with *P*-value < 0.10 in univariate analysis were included in the logistic regression analysis. Logistic regression analysis was performed in two steps. In the first step, the risk factors with *P*-value < 0.10 in univariate analysis were first included using stepwise backward maximum likelihood ratio method with the inclusion criterion of 0.05, exclusion criterion of 0.10, and test level (α) of 0.05. Any continuous variables included in the first step logistic regression analysis model were converted to categorical variables based on clinical experience and literature reports. The second step of logistic regression included all the independent risk factors determined by the first step of logistic regression using the enter method with the inclusion criterion of 0.05, exclusion criterion of 0.10, and test level (α) of 0.05. In order to facilitate clinical application, the logistic regression coefficient (β) of each risk factor was transformed, i.e., the regression coefficient of each risk factor was divided by the smallest regression coefficient (βm) at the same time, and the new regression coefficient after the transformation was rounded to the integer for value assignment to obtain the score of this risk factor (7).

### Evaluation of the Predictive Scoring System

#### Accuracy Evaluation of the Predictive Scoring Model

The accuracy (predictive ability) of the predictive scoring model was evaluated by receiver operating characteristic (ROC) curve analysis; the predictive ability was classified into four levels according to the area under the curve (AUC): (1) AUC≤0.5, no predictive value at all; (2) 0.5 ≤ AUC < 0.7, average predictive ability; (3) 0.7 ≤ AUC < 0.9, high predictive ability; (4) AUC ≥ 0.9, very high predictive ability.

#### Consistency Evaluation of the Predictive Scoring Model

The consistency of the predictive scoring model was evaluated by the Hosmer-Lemeshow test. *P*-value < 0.05 was considered indicative of poor consistency of the predictive scoring model while *P*-value > 0.05 was considered indicative of good consistency.

#### Internal Validation of the Predictive Scoring Model

Internal validation of the established predictive scoring model was performed using the 1,000 bootstrapping sample method, which entails creation of new random datasets (bootstrapping sample datasets) from the original dataset by resampling the sample data with replacement. The distribution of statistics of the bootstrapping sample datasets will be approximately equal to the distribution of the original sample statistics (8). In the present study, the C-index and 95% confidence interval (CI) across the bootstrapping sample datasets were used to evaluate the stability of the established predictive scoring model. These procedures were implemented using the boot package in R (version = 3.6.3).

## Results

### Baseline Data of IAI Patients

Of the 417 IAI patients included in this study, 109 (26.1%) were female; the mean age of patients was 57.1 ± 18.9 years. There were 75 (18.0%) patients with a history of hypertension, 56 (13.4%) patients with coronary artery disease, 37 (8.9%) patients with diabetes mellitus, 72 (17.3%) patients with pneumonia, 190 (45.6%) patients with cancer, 21 (5.0%) patients with hepatic cirrhosis, 26 (6.2%) patients with hypoproteinemia, and 20 (4.8%) patients with chronic renal insufficiency. In our cohort, 163 (39.1%) patients underwent abdominal surgery, and 16 (3.8%) patients received organ transplantation. 197 (47.2%) patients were treated with broad-spectrum antibiotics alone, 158 (37.9%) were treated with a combination of broad-spectrum antibiotics and nitroimidazole drugs, and 62 (14.9%) patients were treated with nitroimidazole drugs alone. A total of 53 (12.7%) patients died during hospitalization.

### Analysis of Risk Factors for In-hospital Death of IAI Patients

On univariate analysis, older age (*P* = 0.018), high APACHE II score (*P* = 0.005), high SOFA score (*P* < 0.001), history of hypertension (*P* = 0.013), pneumonia (*P* < 0.001), abdominal surgery (*P* = 0.002), hypoproteinemia (*P* = 0.002), chronic renal insufficiency (*P* < 0.001), and combination antibiotic therapy (*P* = 0.012) were associated with in-hospital death of IAI patients (Table 1).

**Table 1:** Univariate analysis of in-hospital death in patients with IAI.

**Variables**	**Total**	**Survival**	**Death**	***P*-value**
	**(*n* = 417)**	**(*n* = 364)**	**(*n* = 53)**	
Age, years	57.1 ± 18.9	56.3 ± 18.7	62.9 ± 19.6	**0.018**
≥60	199 (47.7)	168 (46.2)	31 (58.5)	**0.093**
<60	218 (52.3)	196 (53.8)	22 (41.5)	
Body mass index	22.2 ± 4.0	22.3 ± 3.9	21.4 ± 4.7	0.216
APACHE II score	7.2 ± 3.9	7.0 ± 3.8	8.6 ± 4.4	**0.005**
SOFA score	1.0 (0.0–3.0)	1.0 (0.0–2.0)	2.0 (1.0–4.5)	**0.000**
**Gender**				
Male	308 (73.9)	268 (73.6)	40 (75.5)	0.775
Female	109 (26.1)	96 (26.4)	13 (24.5)	
Hypertension	75 (18.0)	59 (16.2)	16 (30.2)	**0.013**
Diabetes mellitus	37 (8.9)	30 (8.2)	7 (13.2)	0.296
Coronary heart disease	56 (13.4)	49 (13.5)	7 (13.2)	0.960
Pneumonia	72 (17.3)	53 (14.6)	19 (35.8)	**0.000**
Abdominal surgery	163 (39.1)	132 (36.3)	31 (58.5)	**0.002**
Cancer	190 (45.6)	162 (44.5)	28 (52.8)	0.256
Hepatic cirrhosis	21 (5.0)	17 (4.7)	4 (7.5)	0.324
Organ transplantation	16 (3.8)	13 (3.6)	3 (5.7)	0.441
Hypoproteinemia	26 (6.2)	17 (4.7)	9 (17.0)	**0.002**
Chronic renal insufficiency	20 (4.8)	11 (3.0)	9 (17.0)	**0.000**
Types of antibiotics				
Broad-spectrum	197 (47.2)	171 (47.0)	26 (49.1)	
Nitroimidazoles	62 (14.9)	61 (16.8)	1 (1.9)	**0.012**
Combined	158 (37.9)	132 (36.3)	26 (49.1)	

*Unless indicated otherwise, data are presented as the number of patients (%). Bold values are statistically significant P-values*.

**Figure 2:**
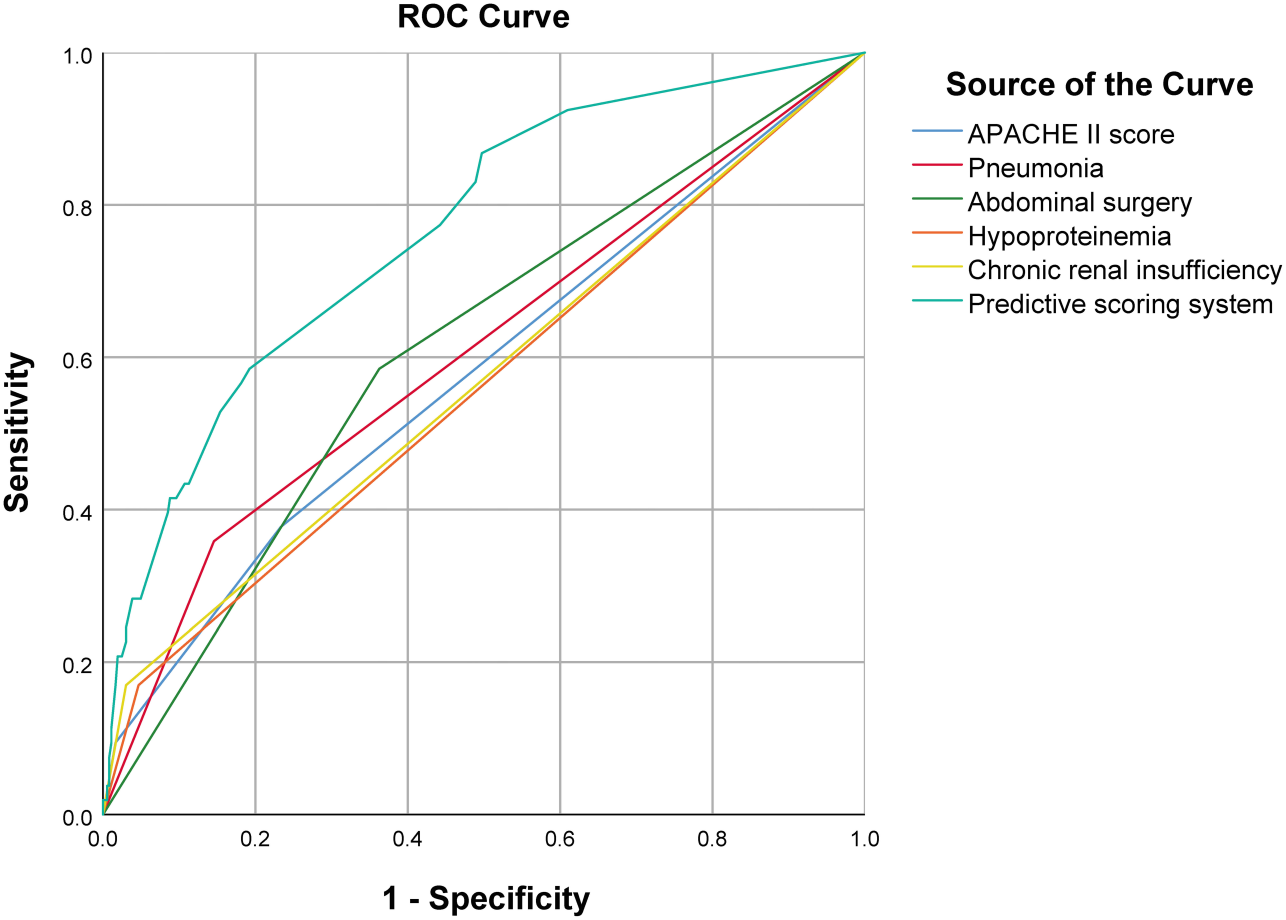
Comparison of ROC curve between predictive scoring system and each independent risk factor.

On multivariate analysis, high APACHE II score (*P* = 0.012), pneumonia (*P* = 0.002), abdominal surgery (*P* = 0.001), hypoproteinemia (*P* = 0.025), and chronic renal insufficiency (*P* = 0.001) were identified as independent risk factors for in-hospital death [AUC: 0.607 (95% CI: 0.522–0.691, *P* = 0.014), 0.606 (95% CI: 0.519–0.694, *P* = 0.002), 0.611 (95% CI: 0.529–0.693, *P* = 0.002), 0.562 (95% CI: 0.473–0.650, *P* = 0.021), and 0.570 (95% CI: 0.481–0.659, *P* = 0.008), respectively]. The AUC of the composite index incorporating these five risk factors was 0.778 (95% CI: 0.711–0.845, *P* < 0.001), and the *P*-value of the Hosmer-Lemeshow test was 0.458. The predictive power of the composite index for in-hospital death of IAI patients was higher than that of the individual risk factors (Figure 2; Tables 2, 3).

**Table 2:** First step of logistic regression analysis of significant variables related to in-hospital death in patients with intra-abdominal infection.

**Variables**	**β**	**SE**	**Wald**	**OR**	**95% CI**	***P*-value**
APACHE II score	0.089	0.040	5.028	1.093	1.011–1.181	0.025
Pneumonia	1.036	0.352	8.668	2.819	1.414–5.618	0.003
Abdominal surgery	1.229	0.337	13.292	3.417	1.765–6.615	0.000
Hypoproteinemia	1.230	0.510	5.827	3.422	1.260–9.294	0.016
Chronic renal insufficiency	1.644	0.533	9.528	5.177	1.822–14.706	0.002
Constant	−3.877	0.460	70.997	0.021		0.000

*SE; standard error; OR, odds ratio; CI, confidence interval*.

**Table 3:** Second step of logistic regression analysis of risk factors of in-hospital death in patients with intra-abdominal infection.

**Variables**	**β**	**SE**	**Wald**	**OR**	**95% CI**	***P*-value**
APACHE II score						
0–9			6.921			0.031
10–15	0.492	0.370	1.766	1.636	0.792–3.380	0.184
≥16	1.735	0.698	6.170	5.668	1.442–22.277	0.013
Pneumonia	1.074	0.350	9.440	2.928	1.475–5.809	0.002
Abdominal surgery	1.127	0.329	11.760	3.085	1.620–5.874	0.001
Hypoproteinemia	1.067	0.510	4.372	2.907	1.069–7.902	0.037
Chronic renal insufficiency	1.810	0.522	12.041	6.109	2.198–16.978	0.001
Constant	−3.184	0.316	101.537	0.040		0.000

*SE, standard error; OR, odds ratio; CI, confidence interval*.

### Establishment of the Scoring System for Predicting the Risk of In-hospital Death in IAI Patients

**Figure 3:**
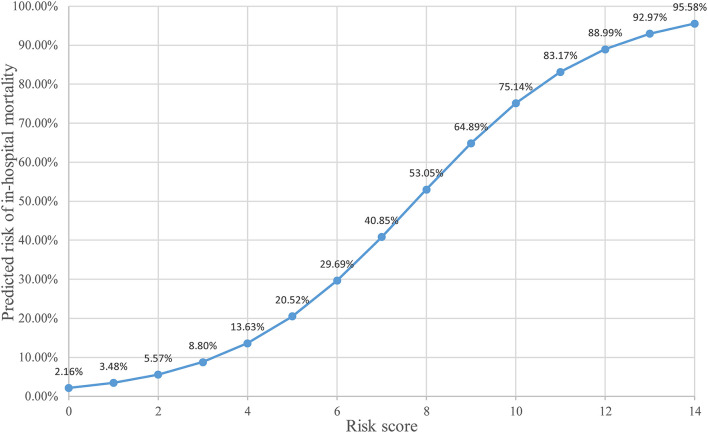
Different risk score correspond to different risk prediction probabilities.

**Figure 4:**
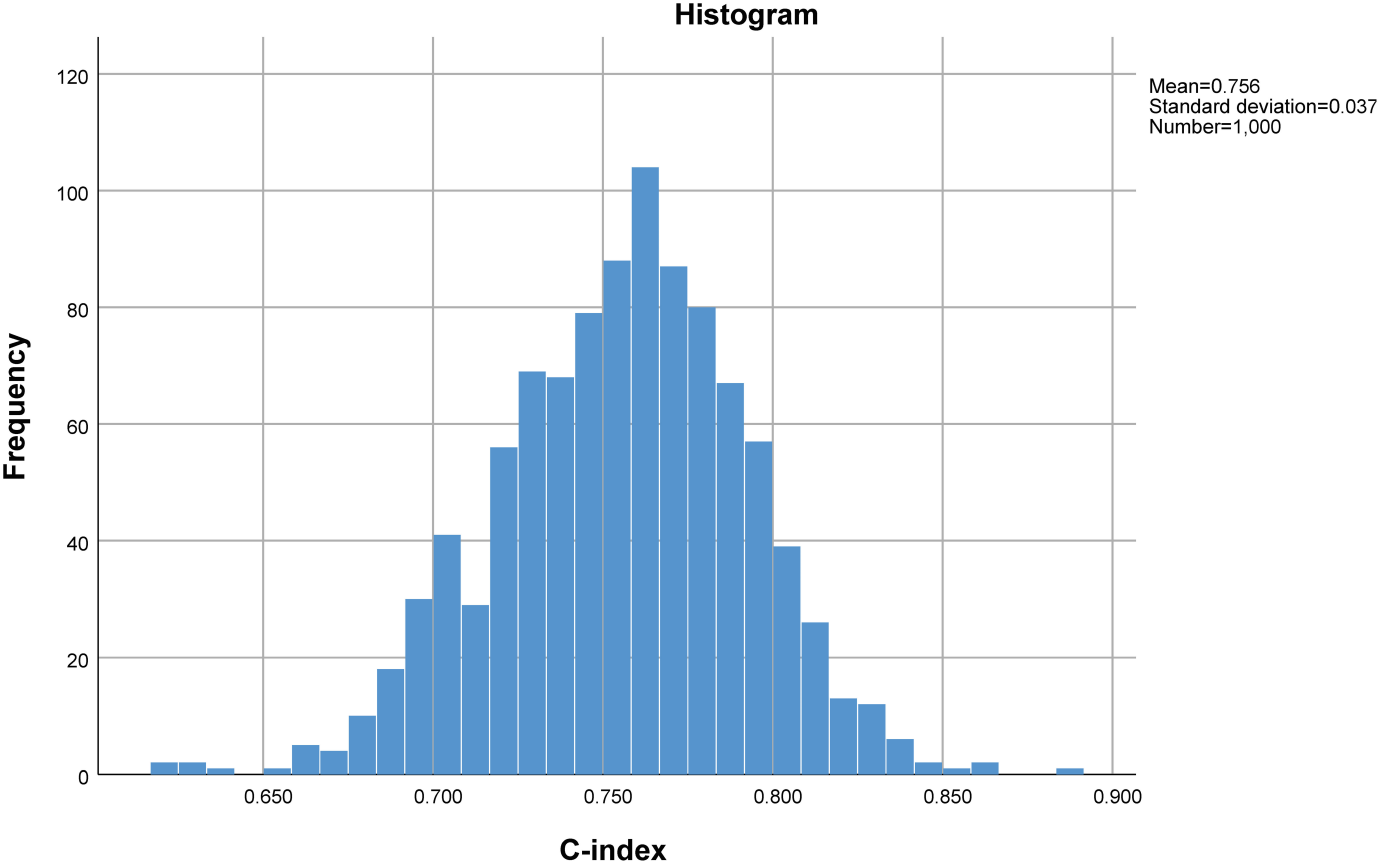
C-index of internal validation of 1,000 bootstrapping sample.

The predictive scoring system (0–14 points) for predicting the risk of in-hospital death of IAI patients was established by re-assigning each risk factor based on the logistic regression coefficient: APACHE II score (10–15 score, 1 point; >15 score, 4 points); pneumonia (2 points), abdominal surgery (2 points), hypoproteinemia (2 points), and chronic renal insufficiency (4 points). The risk of in-hospital death increased with increase in the risk scores. Internal validation of 1,000 bootstrapping sample showed relatively high discriminative ability of the predictive scoring system (C-index = 0.756, 95% CI: 0.753–0.758) (Figures 3, 4; Table 4).

**Table 4:** Risk scores corresponding to different risk factors for in-hospital death in patients with intra-abdominal infection.

**Risk factors**	**Risk score**
APACHE II score	
0–9	0
10–15	1
≥16	4
Pneumonia	
No	0
Yes	2
Abdominal surgery	
No	0
Yes	2
Hypoproteinemia	
No	0
Yes	2
Chronic renal insufficiency	
No	0
Yes	4
Total score	0–14

In order to make the predictive scoring system more convenient for clinical application, the risk scores were divided into three classes based on the clinical experience and published literature: 0–3 scores as low-risk, corresponding to an average in-hospital death rate of 8.50%; 4–7 scores as intermediate-risk, corresponding to an average in-hospital death rate of 33.3%; 8–14 scores as high-risk, corresponding to an average in-hospital death rate of 57.1% (Table 5).

**Table 5:** Three risk classification of the predictive score system.

**Score classification**	**Total score**	**Mortality (%)**	**Risk classification**
I	0–3	8.50	Low
II	4–7	33.33	Moderate
III	8–14	57.14	High

## Discussion

In this study, high APACHE II score, pneumonia, abdominal surgery, hypoproteinemia, and chronic renal insufficiency were identified as independent risk factors for in-hospital death of patients with IAI. The predictive scoring system incorporating these factors reliably predicted the risk of in-hospital death, and the predictive ability was superior to that of the individual risk factors. The predictive scoring system showed high discriminative ability and good consistency. The scoring system can facilitate rapid risk assessment of patients with IAI and help clinicians and patient's families in making reasonable decisions.

The treatment of IAI is extremely challenging. Despite the use of combination antibiotic therapy, the overall morbidity and mortality rates remain high due to proliferation of multi-drug resistant bacteria worldwide (5, 9–11). In a single-center retrospective study of patients with IAI spanning 10 years (*n* = 2,049), patients who received organ transplantation had a significantly higher mortality rate than non-transplant patients (14.0% vs. 8.4%, *P* = 0.008). Furthermore, organ transplant recipients with resistant infections had significantly higher mortality rates than those with non-resistant infections (28.0% vs. 9.5%, *P* = 0.001) (12). These results seem to suggest that the immunosuppressive state due to organ transplantation may not directly increase the mortality risk in IAI patients, but rather increase the risk of infection with drug-resistant bacteria, which in turn increases the risk of death. In the present study, none of the 16 organ transplant recipients were infected with drug-resistant bacteria, and organ transplantation was not identified as a risk factor for in-hospital death on univariate analysis. In a study by Luo et al., in addition to infection with drug-resistant bacteria, enterococcal infection was also associated with increased 28-day mortality, in-hospital mortality, and ICU mortality of IAI patients (13). Of note, Luo et al. also found that abdominal surgery (*P* = 0.004) was an independent risk factor for enterococcal infection, while in the present study, abdominal surgery was an independent risk factor for in-hospital death. This may be attributable to the fact that abdominal surgery increases the risk of dissemination of enterococci from the intestine to the peritoneal cavity, and enterococcal infection further increases the risk of death of IAI patients. Therefore, use of antibiotics covering enterococci may be a reasonable choice for IAI patients with a high risk of enterococcal infection (14, 15).

APACHE II score and SOFA score are widely used for prognostic assessment of critically ill patients. Especially the APACHE II score has been shown to exhibit a positive correlation with the severity of sepsis (6, 16, 17). In the present study, both high APACHE II score and high SOFA score were associated with in-hospital death of IAI patients in the univariate analysis; however, on multivariate analysis, only high APACHE II score was an independent risk factor for in-hospital death. This result also suggests that high APACHE II score is a robust predictor of prognosis in critically ill patients. Similarly, in the study by NurBaykara et al., high APACHE II score of patients with infection at ICU admission was an independent predictor of death (18). Pneumonia and IAI are the first and second leading causes of septic shock, respectively; therefore, patients with concomitant IAI and pneumonia are at a significantly higher risk of septic shock, especially elderly patients (1, 2, 18). Xie et al. conducted a nationwide cross-sectional epidemiological survey of ICU patients with sepsis in China and found that advanced age and pneumonia were associated with 90-day mortality (2). This was consistent with our findings.

Nutritional therapy is fundamental to the treatment of patients with severe infection and can directly affect the prognosis of patients with IAI. A study by Rungsakulkij et al. showed that hypoproteinemia before abdominal surgery was associated with an increased risk of postoperative IAI (19). Moreover, according to a systematic review conducted by Alharbi et al., hypoproteinemia increases the risk of peritonitis in patients undergoing peritoneal dialysis (20). In previous studies, hypoalbuminemia was found to be a predictor of source control failure in IAI patients (6). In our study, hypoproteinemia was an independent risk factor for in-hospital death of IAI patients. Saucedo-Moreno et al. also found that hypoproteinemia increased the risk of death of IAI patients (21). In addition, a meta-analysis by Wiedermann et al. suggested that hypoalbuminemia is a risk factor for acute kidney injury in ICU patients, thereby increasing the risk of subsequent death (22). Similar to the above study, Blot et al. found that malnutrition (low BMI) also increased the risk of death of IAI patients (5). Chronic renal insufficiency, on the other hand, can lead to chronic malnutrition. This also explains the identification of chronic renal insufficiency as an independent risk factor for in-hospital death in this study.

Although several studies have analyzed risk factors for death in IAI patients, no predictive scoring system has been established to assess the risk of in-hospital death in these patients. Treatment of critically ill patients requires continuous monitoring and individualized assessment, and the absence of quantifiable risk factors hinders the prognostic assessment of these patients. The predictive scoring system established in this study can better predict the risk of in-hospital death of IAI patients. We classified the predictive scoring into low-risk, intermediate-risk, and high-risk categories based on clinical experience. This scoring system is a convenient tool for rapid risk assessment of IAI patients, which can help physicians and patients' families in making informed clinical decisions.

Some limitations of our study should be acknowledged. First, this was a single-center retrospective study, which limits the generalizability of the findings. This study only performed internal validation. The scoring system has not yet been externally validated in other centers. Second, this study focused on the risk factors for death during hospital stay and did not address the risk factors of death after discharge. Therefore, the conclusions of this study can only provide a theoretical reference for hospitalized IAI patients. Third, although the number of cases included in this study was relatively large compared with other domestic and overseas studies, the ability to detect potential risk factors was limited due to the relatively low overall mortality rate. Fourth, this study lacked etiological (bacterial) culture and drug susceptibility data; therefore, the effect of drug-resistant bacteria on the risk of death could not be evaluated. In the published literature, the reported incidence of drug-resistant bacteria in IAI is as high as 13.9–26.3%, and pathogen resistance has been widely confirmed to increase mortality of IAI patients (5, 11, 23). Future prospective studies should include etiological data and drug susceptibility profile of pathogens in the analysis.

## Conclusions

In this study, high APACHE II score, pneumonia, abdominal surgery, hypoproteinemia, and chronic renal insufficiency were independent risk factors for in-hospital death of patients with IAI. The predictive scoring system composed of these factors could reliably predict the risk of in-hospital death of patients with IAI, thus providing a theoretical reference for clinicians to develop individualized treatment strategies. However, external validation of the predictive scoring system in a multi-center study is required to further evaluate its predictive performance.

## Data Availability Statement

The raw data supporting the conclusions of this article will be made available by the authors, without undue reservation.

## Ethics Statement

The studies involving human participants were reviewed and approved by the Ethics Committee of the General Hospital of the Southern Theater Command. Written informed consent for participation was not required for this study in accordance with the national legislation and the institutional requirements.

## Author Contributions

GX, HL, and ZL designed the study. GX and HL drafted the article. BJ and ZL critically revised the manuscript and approved the final version of the manuscript on behalf of all authors. All authors contributed to the article, data acquisition, statistical analysis, interpretation of data, and approved the submitted version.

## Funding

This work was supported by grants from the National Natural Science Foundation of China (NO. 82072143) and grants from the PLA Logistics Research Project of China (18CXZ030 and BLJ20J006).

## Conflict of Interest

The authors declare that the research was conducted in the absence of any commercial or financial relationships that could be construed as a potential conflict of interest.

## Publisher's Note

All claims expressed in this article are solely those of the authors and do not necessarily represent those of their affiliated organizations, or those of the publisher, the editors and the reviewers. Any product that may be evaluated in this article, or claim that may be made by its manufacturer, is not guaranteed or endorsed by the publisher.

## Abbreviations

IAI: Intra-abdominal infection
APACHE II: Acute Physiology and Chronic Health Evaluation II
SOFA: Sequential Organ Failure Assessment
AUC: the area under the receiver operating characteristic curve.

## References

[1] Sakr Y Jaschinski U Wittebole X Szakmany T Lipman J Ñamendys-Silva SA. Sepsis in intensive care unit patients: worldwide data from the intensive care over nations audit. Open Forum Infect Dis. (2018) 5:ofy313. 10.1093/ofid/ofy313 30555852 PMC6289022

[2] Xie J Wang H Kang Y Zhou L Liu Z Qin B. The epidemiology of sepsis in chinese ICUs: a national cross-sectional survey. Crit Care Med. (2020) 48:e209–18. 10.1097/CCM.0000000000004155 31804299

[3] Mazuski JE Tessier JM May AK Sawyer RG Nadler EP Rosengart MR. The surgical infection society revised guidelines on the management of intra-abdominal infection. Surg Infect. (2017) 18:1–76. 10.1089/sur.2016.261 28085573

[4] Sartelli M Chichom-Mefire A Labricciosa FM Hardcastle T Abu-Zidan FM Adesunkanmi AK. The management of intra-abdominal infections from a global perspective: 2017 WSES guidelines for management of intra-abdominal infections. World J Emerg Surg. (2017) 12:29. 10.1186/s13017-017-0141-6 28702076 PMC5504840

[5] Blot S Antonelli M Arvaniti K Blot K Creagh-Brown B de Lange D. Epidemiology of intra-abdominal infection and sepsis in critically ill patients: “AbSeS”, a multinational observational cohort study and ESICM Trials Group Project. Intensive Care Med. (2019) 45:1703–17. 10.1007/s00134-019-05819-3 31664501 PMC6863788

[6] Solomkin JS Mazuski JE Bradley JS Rodvold KA Goldstein EJ Baron EJ. Diagnosis and management of complicated intra-abdominal infection in adults and children: guidelines by the surgical infection society and the infectious diseases society of America. Clin Infect Dis. (2010) 50:133–64. 10.1086/649554 20034345

[7] Duan G Yang P Li Q Zuo Q Zhang L Hong B. Prognosis predicting score for endovascular treatment of aneurysmal subarachnoid hemorrhage: a risk modeling study for individual elderly patients. Medicine. (2016) 95:e2686. 10.1097/MD.0000000000002686 26886607 PMC4998607

[8] Hoshino J. Introduction to clinical research based on modern epidemiology. Clin Exp Nephrol. (2020) 24:491–99. 10.1007/s10157-020-01870-3 32212004 PMC7248022

[9] Mazuski JE Gasink LB Armstrong J Broadhurst H Stone GG Rank D. Efficacy and safety of ceftazidime-avibactam plus metronidazole versus meropenem in the treatment of complicated intra-abdominal infection: results from a randomized, controlled, double-blind, phase 3 program. Clin Infect Dis. (2016) 62:1380–89. 10.1093/cid/ciw133 26962078 PMC4872289

[10] De Pascale G Carelli S Vallecoccia MS Cutuli SL Taccheri T Montini L. Risk factors for mortality and cost implications of complicated intra-abdominal infections in critically ill patients. J Crit Care. (2019) 50:169–76. 10.1016/j.jcrc.2018.12.001 30553184

[11] Liu J Zhang L Pan J Huang M Li Y Zhang H. Risk factors and molecular epidemiology of complicated intra-abdominal infections with carbapenem-resistant enterobacteriaceae: a multicenter study in China. J Infect Dis. (2020) 221:S156–63. 10.1093/infdis/jiz574 32176797

[12] Swenson BR Metzger R Hedrick TL McElearney ST Evans HL Smith RL. Choosing antibiotics for intra-abdominal infections: what do we mean by “high risk”. Surg Infect. (2009) 10:29–39. 10.1089/sur.2007.041 19226202 PMC2996818

[13] Luo X Li L Xuan J Zeng Z Zhao H Cai S. Risk factors for enterococcal intra-abdominal infections and outcomes in intensive care unit patients. Surg Infect. (2021) 22:845–53. 10.1089/sur.2020.417 33769911

[14] Dever JB Sheikh MY. Review article: spontaneous bacterial peritonitis–bacteriology, diagnosis, treatment, risk factors and prevention. Aliment Pharmacol Ther. (2015) 41:1116–31. 10.1111/apt.13172 25819304

[15] Zhang J Yu WQ Chen W Wei T Wang CW Zhang JY. Systematic review and meta-analysis of the efficacy of appropriate empiric anti-enterococcal therapy for intra-abdominal infection. Surg Infect. (2021) 22:131–43. 10.1089/sur.2020.001 32471332

[16] Yilmaz TU Kerem M Demirtaş CY Pasaoglu O Taşcilar O Sakrak O. Increased resistin levels in intra-abdominal sepsis: correlation with proinflammatory cytokines and acute physiology and chronic health evaluation (APACHE) II scores. Sultan Qaboos Univ Med J. (2014) 14:e506–12. 25364554 PMC4205063

[17] Huang CT Ruan SY Tsai YJ Ku SC Yu CJ. Clinical trajectories and causes of death in septic patients with a low APACHE II score. J Clin Med. (2019) 8:1064. 10.3390/jcm8071064 31330785 PMC6678558

[18] Baykara N Akalin H Arslantaş MK Hanci V Çaglayan Ç Kahveci F. Epidemiology of sepsis in intensive care units in Turkey: a multicenter, point-prevalence study. Crit Care. (2018) 22:93. 10.1186/s13054-018-2013-1 29656714 PMC5901868

[19] Rungsakulkij N Vassanasiri W Tangtawee P Suragul W Muangkaew P Mingphruedhi S. Preoperative serum albumin is associated with intra-abdominal infection following major hepatectomy. J Hepatobiliary Pancreat Sci. (2019) 26:479–89. 10.1002/jhbp.673 31532926 PMC6899963

[20] Alharbi MA. Low serum albumin a predictor sign of the incidence of peritoneal dialysis-associated peritonitis? A quasi-systematic review. Saudi J Kidney Dis Transpl. (2020) 31:320–34. 10.4103/1319-2442.284006 32394904

[21] Saucedo-Moreno EM Fernández-Rivera E Ricárdez-García JA. Hypoalbuminemia as a predictor of mortality in abdominal sepsis. Cir Cir. (2020) 88:481–84. 10.24875/CIRU.20001712 32567597

[22] Wiedermann CJ Wiedermann W Joannidis M. Hypoalbuminemia and acute kidney injury: a meta-analysis of observational clinical studies. Intensive Care Med. (2010) 36:1657–65. 10.1007/s00134-010-1928-z 20517593 PMC7728653

[23] Labricciosa FM Sartelli M Abbo LM Barbadoro P Ansaloni L Coccolini F. Epidemiology and risk factors for isolation of multi-drug-resistant organisms in patients with complicated intra-abdominal infections. Surg Infect. (2018) 19:264–72. 10.1089/sur.2017.217 29298133

